# Case of Removal of Both Testes for Spermatorrhœa, by a Botanic Physician — Case of Death from Inhalation of Chloroform

**Published:** 1859-03

**Authors:** La Fayette Avery

**Affiliations:** South Otselic


ART. II.—*Case of Removal of both Testes for Spermatorrhœa, by a Botanic Physician—Case of Death from Inhalation of Chloroform.* By LA FAYETTE AVERY, M. D., South Otselic.

An article published in the December number on Spermatorrhœa, and one in the January number, on the Use of Chloroform, have prompted me to communicate the following items in my experience. I had supposed that I had the minutes of the only case of spermatorrhœa ever treated by the process of castration; and, although I was in error on that point, the following case may yet be thought worth publishing:

In the summer of 1857 a messenger came to my office, and gave me a very pressing invitation to ride a distance of three miles, and assist a botanic practitioner in removing the testicles of a man who was suffering from the above mentioned disease, and had expressed his desire to be dealt with in that way. The patient, a man of about thirty-five years of age, was a large, bony fellow, and otherwise healthy. He had been the rounds of all the irregular practitioners of the county, without obtaining relief, and now somewhat feeble, cold, and bloodless, had desired to be rid of the troublesome cause of his nocturnal pollutions. I declined the honor of being present during the performance of the proposed surgery; but a neighboring physician, who had also been invited to attend, allowed his desire for amusement to get the better of his sense of professional propriety, and he consented to be present, as a looker on, in the matter; and from him I have learned some of the particulars of the operation.

The patient, after all things were ready for the operation, was allowed a good glass of whiskey, to assist his firmness, and then requested to doff his pantaloons, and lie upon his face across a bed, with his feet standing upon the floor. The operator then seized the scrotum with his left hand, and drawing it backwards, after the manner of our farmers, in similar performances, made the incision, and after a tedious dissection, succeeded in extirpating one of the glands. The patient, in the mean time, complained much of the dullness of the knife, and demanded a respite, and some whiskey, which were allowed him. The botanic then borrowed a knife of keener edge from the physician in attendance, and removed the remaining testicle with more celerity.

I know but little of the after history of the case, except that an abscess occurred on one side of the scrotum, notwithstanding which, his recovery was tolerably rapid. For a while, things went on swimmingly with the patient. His seminal emissions were much less; his blood was of better quality, and his general health improved. He congratulated himself all around: the disturbers of his peace he had preserved in spirits, and placed upon a shelf at the foot of his bed; warmth and health came back to him, and increased upon him every day, but what he most felicitated himself upon, was the discovery of a shorter road to divorce from matrimony than any hitherto known to the courts, for his wife left his bed and board on the instant.

His present condition, as I learn from inquiry, is as follows: General health partially restored, and he is able to do a moderate day's work. There is still spermatorrhœa, and he much regrets the loss of his seminal glands. He can look no one in the face, and says he would give one thousand dollars to have his manhood back again.

I observe, with pleasure, that you have turned your attention to the subject of anæsthetics, and, in a measure, criticise the use of chloroform. I imagine that a small part of the misfortunes resulting from the use of that article have yet seen the light, and that when the lapse of time shall have removed the fear of personal discredit in communicating the whole truth, by those who have had experience with it, a fearful picture will be presented. Having, myself, put a fellow being out of existence with the article, I am anxious to bear my testimony against its use, by relating the case.

In the fall of 1850 I was requested to remove the leg of Waldo Ferris, who had been under the treatment of a quack, for an injury of the knee, which had resulted badly, rendering an amputation at the thigh indispensable. Ferris had been a strong man, and had indulged in intemperate habits, but now, at the age of forty years, was free from disease, except the injured limb. He was considerably emaciated, being much worn down by his disease, yet his appetite was good, and he smoked his pipe, and conversed freely,

He was prepared for the operation when I arrived, and had an experienced dentist on hand, to administer him chloroform. I advised against its use, on account of his debility, but finally yielded to the solicitations of the patient, and allowed it to be given.

He was placed upon the table, and assisted by Drs. Jaimeson and Ford, I proceeded to the business before us. Some chloroform was poured upon a silk handkerchief and applied to the patient's mouth and nose without intermission, for, as near as I could judge, a minute, when I noticed a labored breathing on the part of the patient, and at the same time Dr. Jaimeson mentioned a partial failure of the pulse. I immediately ordered the dentist to desist with the chloroform, and gave a swallow of brandy, which improved the pulse, but still the patient breathed in a stertorous manner, and his face was congested and purple. No more chloroform was given. I hesitated a moment, and then proceeded to remove the leg and dress the stump, the patient continuing insensible, and snoring; face still of a purplish hue, and pulse feeble and frequent. We then put him in bed, and used all the means we were master of to rally him, but without avail. He died in about twenty-five minutes from the time he first inhaled the chloroform, his face purple to the last.

The dentist was a reliable man, and he assured me that he had previously used of the same chloroform, and had found it to be good. On examining the vial containing the article, not more than two drachms were wanting, and the dentist said the vial was not full when first uncorked.

Now, this patient evidently died of the chloroform, as the reflections of any physician will satisfy him; and I desire to know what right a surgeon has to use an article that sometimes produces such effects as this. I, for one, have parted company with the poisonous thing, and am very confident that I shall not renew my acquaintance.

